# Prevalence of urinary schistosomiasis amongst school-age children in Abuja, Nigeria

**DOI:** 10.11604/pamj.2026.53.176.51363

**Published:** 2026-04-28

**Authors:** Dan Apagu Gadzama, Julia Arthur, Ben Gold, Dolapo Clement, Sadiq Abdulrahaman, Jenny Momoh

**Affiliations:** 1 Department of Community Medicine and Public Health, Nile University of Nigeria, Abuja, Nigeria,; 2 University of Westminster, London, United Kingdom,; 3 World Health Organization, Federal Capital Territory, Abuja, Nigeria

**Keywords:** Urogenital schistosomiasis, prevalence, school-aged children, Abuja

## Abstract

**Introduction:**

urogenital schistosomiasis affects an estimated 240 million people globally, with ~90% of cases occurring in Africa. Nigeria bears a substantial burden, with ~29 million people affected. This study assessed the community-level prevalence of urinary schistosomiasis among school-aged children in Abuja.

**Methods:**

a descriptive cross-sectional survey was conducted in the Federal Capital Territory (FCT) in April 2025. School-aged children provided urine samples, which were examined using urine filtration.

**Results:**

of 400 participants, 174 (43.5%) were aged 5-9 years, 196 (49.0%) 10-14 years, and 30 (7.5%) 15-19 years. Microscopy identified 124 (31.0%) positive cases; males and females accounted for 58.1% and 41.9% of infections, respectively. Children aged 10-14 years were most affected.

**Conclusion:**

urinary schistosomiasis remains endemic in Abuja. Sustained public health interventions are required to curb transmission and mitigate long-term complications in children.

## Introduction

Urogenital schistosomiasis affects an estimated 240 million people globally, with ~90% of cases reported in Africa [1,2]. The disease remains prevalent in sub-Saharan Africa, parts of South America and the Caribbean, the Middle East, and areas of Southern Asia, where ~779 million people are at risk of infection [3]. It is endemic in 78 countries, particularly in tropical and subtropical regions with limited access to safe water and poor sanitation [2]. Urogenital schistosomiasis – caused primarily by the trematode *Schistosoma haematobium* – is a neglected tropical disease (NTD) and a leading cause of morbidity. It is often described as the third most important parasitic cause of morbidity and mortality in many developing countries after malaria and intestinal helminthiases [4].

Nigeria carries a substantial burden of urogenital schistosomiasis, with an estimated 29 million people infected and ~101 million at risk [5]. School-aged children constitute a large proportion of those affected [6]. The disease continues to pose a significant public health problem, particularly in rural and peri-urban settings, where it contributes meaningfully to morbidity and, if untreated, to long-term complications [7]. Abuja, Nigeria's Federal Capital Territory (FCT), is not exempt; studies indicate a high prevalence in parts of the city [8,9]. Ongoing surveillance is therefore essential for planning, targeting, and sustaining prevention and control strategies.

Within the FCT, the Abuja Municipal Area Council (AMAC), the FCT Public Health Department, and the Federal Ministry of Health confront considerable challenges related to schistosomiasis, including chronic bladder infections, kidney damage, and bladder cancer, as well as reproductive complications in severe cases. Sensitive diagnostics capable of detecting low-intensity infection are especially important for early case finding and timely treatment. Transmission in central Nigeria is seasonal, and prior surveys from comparable settings have shown high prevalence levels, underscoring the need for regular monitoring to guide control and potential elimination efforts [10,11]. To this end, evaluating infection rates – particularly among preschool and school-aged children who frequently serve as primary reservoirs – is critical for breaking transmission.

### Study aim

This study was designed to describe the prevalence of urinary schistosomiasis among school-aged children in selected communities within AMAC, FCT. By generating up-to-date, community-level data, the study provides an empirical basis for strengthening surveillance, refining control strategies, and informing targeted public health interventions.

## Methods

### Study area

The study was conducted in Nigeria's Federal Capital Territory (FCT), located in the northern region of the country between 9° 4' 20.1504" N and 7° 29' 28.6872" E, with an approximate land area of 7,315 km^2^. The FCT lies slightly west of the national midpoint and north of the confluence of the Niger and Benue Rivers, and is bordered by Kaduna (northwest), Nasarawa (northeast), Kogi (southwest), and Niger (west/northwest). Administratively, it comprises six Area Councils and 62 wards. The capital is predominantly urban, with adjoining semi-urban and rural settlements. Urban areas have greater infrastructure and connectivity (airports, rail, roads), whereas rural and peri-urban areas have limited sanitation and healthcare access. The climate is tropical savannah, with annual rainfall of roughly 1,100-1,600 mm and temperatures ranging from 21-40°C (mean ~26°C). The presence of rivers, streams, and backwaters provides potential snail habitats that serve as intermediate hosts for *Schistosoma spp*. The Abuja Municipal Area Council (AMAC), one of six councils, has 138 villages across 12 wards (Nyanya, Gui, Gwagwa, Kabusa, Gwarinpa, Orozo, Karu, Garki, Karshi, Jiwa, City Centre, Wuse) [12]. Environmental surveys indicate multiple water sources (wells, ponds, backwaters, taps) and numerous communities without reliable tap water [13]. Community-directed praziquantel mass treatment is typically provided annually for school-aged children.

### Study design and site selection

A descriptive cross-sectional survey was conducted in selected rural communities of AMAC (FCT). Schools were chosen from areas previously identified with urogenital schistosomiasis endemicity, based on existing survey reports and local knowledge. Site selection prioritized communities with established or suspected exposure to natural water bodies used for domestic and recreational purposes.

### Sample size and participant selection

The sample size was proportional to the combined size of the target school populations across selected villages. Assuming a 10% prevalence, 95% confidence level, and 5% alpha error, the calculated sample was 428 children, proportionally allocated to each village according to population. Inclusion criteria were age 4-18 years, residence in the community for =1 year, and parental consent with child assent where applicable. Children who had received praziquantel within the previous 6 months were excluded to avoid misclassification of recent treatment effects. A total of 400 school-aged children ultimately participated.

### Data collection and urine sampling

Urine collection was performed on a single day in each village between 10:30 a.m. and 2:00 p.m. Children provided 10 mL midstream urine specimens into clean, labelled, sealed universal containers. Samples were kept at approximately 4°C in ice packs and transported to the laboratory the same day for analysis.

### Parasitological examination

Urine specimens were examined using the urine filtration technique under standard operating procedures. A 10 mL aliquot was passed through a filter membrane; filters were stained with Lugol's iodine and read by light microscopy at 100x and 400x magnifications. Counts were standardized to eggs per 10 mL of urine. Infection intensity was classified as light or heavy (=50 eggs/10 mL) in line with the working threshold used in the study's laboratory protocol. All slides were read by trained personnel; equivocal findings were checked by a second reader for internal consistency.

### Variables and measures

***Outcome:*** presence of *Schistosoma haematobium* eggs (positive/negative).

***Infection intensity:*** light vs heavy (=50 eggs/10 mL). *Predictors:* age (categorized: 5-9, 10-14, 15-19 years), sex, educational level (Primary 1-Primary 4; Primary 5-Junior Secondary 2; Junior Secondary 3-Senior Secondary 3), and duration of residence (<1 year; 1-5 years; 6-10 years; >10 years).

***Descriptives:*** frequencies, percentages, and mean age (years).

### Data management and statistical analysis

Field data were recorded on standardized forms and entered into Excel before import into the Statistical Package for Social Sciences (SPSS version 25), for analysis. Descriptive statistics summarized the sample (frequencies, percentages, means). Chi-square tests assessed bivariate associations between infection status and sociodemographic characteristics (age, sex, education, duration of residence). Multiple logistic regression was then fitted to identify independent predictors of infection, entering age, sex, education, and duration of residence simultaneously. Results were reported as regression coefficients (β), adjusted odds ratios (aOR), and 95% confidence intervals (CI). Statistical significance was set at p < 0.05.

### Quality assurance

All field workers were briefed on sampling, labelling, and cold chain transport. Laboratory staff followed a written standard operating procedure for filtration, staining, and microscopy, with periodic spot checks for consistency. Data entry was double-checked for completeness and logic (range checks for age, internal consistency across demographic fields).

### Ethical considerations

Ethical approval was obtained from the FCT Health Research Ethics Committee (Approval N°: FHREC/2025/01/74/20 03 25). Permissions were obtained from local authorities and school administrations. Written informed consent was obtained from parents/guardians for all child participants, with assent as appropriate to age. Children with positive results were referred for appropriate treatment in collaboration with local primary healthcare services.

## Results

### Urinary schistosomiasis characteristics of the study population

A total of 400 school-aged children participated in the survey. By age distribution, 196 (49.0%) were 10-14 years, 174 (43.5%) were 5-9 years, and 30 (7.5%) were 15-19 years. Males comprised 247 (61.75%) of respondents, while females were 153 (38.25%). Regarding educational level, 132 (33.0%) were in Primary 1-Primary 4, 184 (46.0%) in Primary 5-Junior Secondary 2, and 84 (21.0%) in Junior Secondary 3-Senior Secondary 3. For duration of residence, 30 (7.5%) had lived in their community for less than one year, 78 (19.5%) for 1-5 years, 84 (21.0%) for 6-10 years, and 208 (52.0%) for over 10 years. The mean age was 12 years (Table 1).

**Table 1:** sociodemographic characteristics of school-aged children

Variable	Frequency (n = 400)	Percent (100)
**Age (year)**		
5-9	174	43.5
10-14	196	49.0
15-19	30	7.5
**Mean**	**12 ± 4.08**	
**Gender**		
Male	247	61.75
Female	153	38.25
**Educational level**		
Primary 1 - primary 4	132	33.0
Primary 5 - Junior secondary 2	184	46.0
Junior secondary 3- senior secondary 3	84	21.0
**Stay duration**		
Less than a year	30	7.5
1-5 Years	78	19.5
6-10 Years	84	21.0
Over 10 years	208	52.0

### Prevalence of urinary schistosomiasis by sex and age group

Overall, 124 of 400 children were positive for *Schistosoma haematobium*, yielding a prevalence of 31.0%. Prevalence was higher in males (58.1%) than in females (41.9%). By age group, infection was most common among children aged 10-14 years (58.1%), followed by those aged 5-9 years (32.2%); the 15-19 year group showed the lowest proportion of infections (9.7%). A chi-square test indicated no significant association between sex and infection status (χ^2^ = 1.033; p = 0.309). In contrast, age was significantly associated with infection (χ^2^ = 9.371; p = 0.009), indicating that infection likelihood varied across age categories. These results are summarized in Table 2.

**Table 2:** sex and age prevalence of urinary schistosomiasis in FCT

Sex	Number examined	Number infected	Prevalence (%)
Male	247	72	58.1
Female	153	52	41.9
**Total**	**400**	**124**	**31.0**
X^2^, p value			1.033 (0.309)
**Age group**			
5-9	174	40	32.2
10-14	196	72	58.1
15-19	30	12	9.7
**Total**	**400**	**124**	**31.0**
X^2^, p-value			9.371 (0.009)

### Association between sociodemographic characteristics and infection status

Table 3 shows the chi-square analysis of sociodemographic factors against infection status. A significant association was found for age (χ^2^ = 9.371; df = 2; p = 0.009), with the 10-14 year group contributing the highest proportion of positives (36.7% of that stratum) compared with 23.0% in the 5-9 year group and 40.0% in the 15-19 year group (noting that this latter group was the smallest by size). No statistically significant associations were observed for gender (χ^2^ = 1.033; df = 1; p = 0.309), educational level (χ^2^ = 1.417; df = 2; p = 0.492), or duration of residence (χ^2^ = 7.461; df = 3; p = 0.059), although the latter approached significance.

**Table 3:** association between sociodemographic characteristics of school children and inspected cases

Variable	Cases Inspected			χ^2^	Df	p-value
	Positive	Negative	Total			
	**n = 124**	**n = 276**	**n = 400**			
**Age (year)**						
5-9	40 (23)	134 (77)	174	9.371	2	*0.009
10-14	72 (36.7)	124 (63.3)	196			
15-19	12 (40)	18 (60)	30			
**Gender**						
Male	72 (29.1)	175 (70.9)	247	1.033	1	0.309
Female	52 (34)	101 (66)	153			
**Educational status**						
Primary 1 - Primary 4	37(28.03)	95 (71.96)	132	1.417	2	0.492
Primary 5 - JSS 2	57(30.98)	127 (69.12)	184			
JSS 3- SSS 3	30 (35.71)	54 ()	84			
**Stay duration**						
Less than a year	5 (16.7)	25 (83.3)	30	7.461	3	0.059
1-5 Years	32 (41)	46 (59)	78			
6-10 Years	22 (26.2)	62 (73.8)	84			
10 Year above	65 (31.3)	143 (68.8)	208			

**Notes:** *Statistically significant at p < 0.05

### Predictors of infection: multiple logistic regression

Multiple logistic regression examining age, gender, educational level, and duration of residence identified age as the only significant predictor of infection. Specifically, increasing age was associated with lower odds of infection (β = -0.474; p = 0.009; adjusted OR = 0.623; 95% CI: 0.437-0.887). The model constant was significant (β = 2.000; p = 0.001; OR = 7.389), reflecting baseline log odds when predictors are zero. Gender (p = 0.585; adjusted OR = 0.884; 95% CI: 0.568-1.376), educational level (p = 0.503; adjusted OR = 0.902; 95% CI: 0.666-1.221), and duration of residence (p = 0.915; adjusted OR = 0.988; 95% CI: 0.795-1.228) were not significant predictors. Full regression outputs are presented in Table 4.

**Table 4:** multiple logistic regression analysis of sociodemographic factors among school children as predictors of the prevalence of urinary schistosomiasis

Predictor variable	B	SE	Wald	p-value	Adj. OR	95% CI for OR	
						Lower	Upper
Constant	2.000	0.598	11.190	0.001	7.389		
Age of School children (overall)	-0.474	0.180	6.899	0.009	0.623	0.437	0.887
Gender of School children (overall)	-0.123	0.226	0.298	0.585	0.884	0.568	1.376
Education Level of School children (overall)	-0.104	0.155	0.448	0.503	0.902	0.666	1.221
Stay duration of School children (overall)	-0.012	0.111	0.011	0.915	0.988	0.795	1.228

β = Regression coefficient; SE = Standard error; Adj. = Adjusted; OR = Odds ratio

### Key points from the results

***Prevalence:*** overall 31.0% (124/400).

***Sex:*** higher infection proportion among males (58.1%) than females (41.9%); not significant (p = 0.309).

***Age:*** highest infection in 10-14-year-olds; significant association with infection (p = 0.009).

***Predictors****:* age independently predicts lower odds of infection with increasing years (adjusted OR 0.623; p = 0.009).

## Discussion

This study confirms that urinary schistosomiasis remains endemic among school-aged children in Abuja, with an overall prevalence of 31.0%. The burden was higher among males (58.1%) than females (41.9%), and age showed a statistically significant association with infection status (*p*= 0.009), with the 10-14-year age group most affected. These patterns are consistent with the recognized epidemiology of *Schistosoma haematobium*, wherein school-aged children are more likely to engage in recreational and domestic water contact that facilitates exposure to infected freshwater snails. The observed sex pattern – males accounting for a larger share of infections – has frequently been attributed to behavior that increases water exposure (e.g., swimming, fishing, water play), although some studies have reported no significant sex differences depending on local context and exposure profiles.

The age-related gradient observed in this cohort aligns with widely reported trends: infection intensifies through late childhood and early adolescence and then falls in later adolescence and adulthood as behaviors change and partial immunity potentially develops. In our data, age was the only independent predictor of infection in multivariable analysis (β = -0.474; p = 0.009; adjusted OR = 0.623; 95% CI: 0.437-0.887), indicating that each additional year of age was associated with lower odds of infection. Importantly, this pattern complements the chi-square findings showing the 10-14 year group as the highest risk stratum. Together, these results underscore the need to prioritize school-based case finding, prevention, and treatment.

Although prevalence was higher among males, the association between sex and infection status was not statistically significant in this sample (p = 0.309). This finding suggests that, while boys may be more represented among positives, sex alone is not a robust determinant of infection when other factors are considered. Similarly, educational level and duration of residence were not significantly associated with infection in bivariate analyses (*p*= 0.492 and p = 0.059, respectively), although the latter approached significance. The near-significant signal for length of residence plausibly reflects cumulative exposure in communities with perennial or seasonal transmission; however, the present data do not establish a definitive relationship. Future analyses with larger samples or finer stratification of exposure variables (e.g., specific water contact behaviors, frequency, and duration) could clarify whether residence time contributes meaningfully to risk.

From a public health standpoint, the 31.0% prevalence found here falls within the broad range reported in Nigerian settings, where ecological heterogeneity, water infrastructure, sanitation, and behavior drive substantial variability in transmission intensity. The concentration of infections in children aged 10-14 years signals the importance of timely, age-targeted interventions, including health education on water contact risk, safe water access, sanitation improvements, and regular praziquantel administration through school platforms. In addition, because light-intensity infections can still contribute to morbidity and sustain transmission, programmatic strategies should incorporate sensitive diagnostics in surveillance and evaluation cycles, especially in settings where prevalence fluctuates seasonally and control programs aim for elimination as a public health problem.

The diagnostic approach used – urine filtration with microscopy – remains a WHO-recommended standard for detecting *S. haematobium* eggs and is appropriate for prevalence estimation in endemic communities. Nonetheless, filtration has reduced sensitivity at lower infection intensities and may miss early or light infections, potentially leading to underestimation of true prevalence. This limitation is particularly relevant where mass drug administration (MDA) or improved water/sanitation interventions have begun to depress intensities. Complementing routine microscopy with more sensitive tools (e.g., antigen detection where feasible) could strengthen surveillance, especially in borderline prevalence areas and during program evaluation phases.

The clinical implications of persistent transmission are substantial. Chronic *S. haematobium* infection contributes to haematuria, dysuria, anaemia, urinary tract pathology, and – over time – bladder and urinary tract complications. The concentration of infection in school-aged children implies ongoing risks to growth, cognition, and educational attainment, in addition to the long-term urological sequelae. The present findings, therefore, reinforce the need for integrated control packages combining MDA, behavior change communication, safe water provision, and sanitation/hygiene measures. Because exposure is often community-wide and shaped by local water sources and practices, community engagement – including with parents, teachers, and traditional leaders – remains essential for sustained impact.

Finally, several limitations warrant consideration. First, the cross-sectional design precludes causal inference regarding predictors of infection and cannot capture seasonal variation directly. Second, reliance on single-day urine sampling may underdetect intermittent egg excretion. Third, the study did not quantify individual water contact behaviors or specific environmental exposures, which could explain residual confounding and help disentangle age- and sex-related risk more precisely. Despite these limitations, the study offers current, community-level evidence from Abuja that can guide programming, surveillance, and targeted interventions in school-aged populations.

In summary, urinary schistosomiasis remains a significant public health concern in Abuja, with the highest burden among 10-14-year-old children and age emerging as the principal predictor of infection. Strengthening school-based control, enhancing sensitive surveillance, and addressing water and sanitation gaps are critical next steps to reduce transmission and long-term morbidity in affected communities.

## Conclusion

This study demonstrates that urinary schistosomiasis remains endemic among school-aged children in Abuja, with an overall prevalence of 31.0%. Infection was most concentrated among 10-14-year-olds, and age emerged as the sole independent predictor of infection, with increasing age associated with reduced odds of positivity. Although a higher proportion of infections occurred among males than females, sex was not significantly associated with infection status; similarly, educational level and duration of residence showed no statistically significant relationships. These findings indicate that routine exposure patterns during late childhood and early adolescence – rather than demographic characteristics alone – are the principal drivers of infection risk in the study communities. Programmatically, the results underscore the need to intensify school-based prevention and control. Priorities include regular praziquantel administration, behavior change communication focused on high-risk water contact, and improvements in safe water access and sanitation. Given the potential for light intensity infections to sustain transmission and cause morbidity, surveillance should incorporate sensitive diagnostic approaches alongside routine microscopy to better capture low burden infections. Finally, because local water use and contact behaviors shape risk, sustained community engagement – including parents, teachers, and local leaders – will be critical to the effectiveness and durability of control efforts in Abuja's school-aged populations.

### What is known about this topic

- Endemicity of urinary schistosomiasis in Abuja, like other parts of Nigeria;
- In many endemic communities, including parts of Abuja, children aged 5-14 years bear the highest burden.

### What this study adds

- True prevalence, including low-intensity infections;
- Gender differences in rate of infection.
